# Dutch Translation, Cultural Adaption, and Validation of the German Pelvic Floor Questionnaire for Pregnant and Postpartum Women

**DOI:** 10.1007/s00192-025-06173-3

**Published:** 2025-06-03

**Authors:** Linde M. L. Titulaer, Viola C. Sandfort, Lidwine B. Mokkink, Annelies L. Pool-Goudzwaard, Marco H. Blanker, Gert-Jan van Baaren, Anna E. Seijmonsbergen-Schermers, Jan-Paul W. R. Roovers, Corine J. M. Verhoeven

**Affiliations:** 1 Department of Midwifery Science, MF G102 d, Amsterdam UMC, Van Der Boechorststraat 7, 1081 BT Amsterdam, The Netherlands; 2 Midwifery Academy Amsterdam Groningen, Inholland, Amsterdam, The Netherlands; 3 Amsterdam Public Health Research Institute, Amsterdam UMC, Amsterdam, The Netherlands; 4 Department of Primary and Long-Term Care, University of Groningen, University Medical Center Groningen, Groningen, The Netherlands; 5 Faculty of Medicine, Leiden University, Leiden University Medical Center, Leiden, The Netherlands; 6 Department of Epidemiology and Data Science, Amsterdam UMC, Vrije Universiteit, Amsterdam, The Netherlands; 7 Amsterdam Movement Sciences, Vrije Universiteit Amsterdam, Amsterdam, The Netherlands; 8 SOMT University of Physiotherapy, Amersfoort, The Netherlands; 9 Department of Obstetrics and Gynaecology, OLVG, Amsterdam, The Netherlands; 10 Department of Obstetrics and Gynaecology, Amsterdam UMC, Amsterdam, The Netherlands; 11 Amsterdam Reproduction and Development Research Institute, Amsterdam UMC, Amsterdam, The Netherlands; 12 Department of Obstetrics and Gynaecology, Maxima Medical Centre, Veldhoven, The Netherlands; 13 Division of Midwifery, School of Health Sciences, University of Nottingham, Nottingham, UK

**Keywords:** Maternal health, Pelvic floor, Postpartum period, Pregnancy, Surveys and questionnaires

## Abstract

**Introduction and Hypothesis:**

Pregnancy and childbirth are important risk factors for developing pelvic floor dysfunction (PFD). A valid and reliable screening instrument is essential for timely identification and treatment of PFD. The German Pelvic Floor Questionnaire for Pregnant and Postpartum women (PFQ-PP) assesses symptoms, severity, and impact on quality of life for the bladder function, bowel function, prolapse, and sexual health domain. However, no similar questionnaire exists in Dutch. This study was aimed at translating, culturally adapting, and validating the German PFQ-PP for use in the Netherlands, resulting in the Dutch PFQ-PP.

**Methods:**

A forward–backward translation method was used to translate and cross-culturally adapt the German PFQ-PP. Content validity was assessed by an expert committee of seven health care professionals who reviewed the translated version, and by using semi-structured cognitive interviews with four pregnant and five postpartum women. For field testing, 202 pregnant and postpartum women completed the questionnaire twice, with a 1-week interval to assess test–retest reliability, measurement error, and known group validity.

**Results:**

The Dutch PFQ-PP demonstrated good content validity both in health care professionals and in pregnant and postpartum women. Intraclass correlation coefficients ranged from 0.82 to 0.92. Standard errors of measurement ranged from 0.38 to 0.60 for the four domains (all domain scores ranging from 0 to 10). The questionnaire discriminated as expected between women suffering from symptoms and those who do not.

**Conclusions:**

The Dutch PFQ-PP is valid and reliable in assessing bladder function, bowel function, prolapse, and sexual health in pregnant and postpartum women.

**Supplementary Information:**

The online version contains supplementary material available at 10.1007/s00192-025-06173-3

## Introduction

Pelvic floor dysfunction (PFD) can significantly affect social, psychological, and physical well-being, reducing quality of life and self-image [1, 2]. PFD is highly prevalent: urinary incontinence (UI) affects 25–45% of all women [3], fecal incontinence (FI) is experienced by 9% [4], and objectified pelvic organ prolapse (POP) is reported to be 36% [5]. Sexual dysfunction is experienced by 44% of women in the general population [6]. The effects of pregnancy and childbirth on the pelvic floor are reflected in the prevalence of PFD in pregnant and postpartum women. The prevalence of UI during pregnancy is estimated to be 41% [7]. Between 6 and 12 months postpartum, 56% of women experience some kind of UI [8]. Moreover, 64% of women reported one or more sexual health issues at 12 months postpartum [9]. The impact of PFD after childbirth is long lasting. Research involving primiparous women 20 years after giving birth showed a prevalence of any PFD of 46.5% [10].

Despite the high prevalence and impact of PFDs among pregnant and postpartum women, only a small proportion seek professional help [8]. Many women consider their symptoms to be a normal part of pregnancy or are unaware of treatment options [9, 11]. Additionally, health care professionals often miss opportunities to address pelvic floor function during prenatal or postpartum check-ups [9, 11, 12]. Early identification and treatment of PFD can potentially prevent these issues from becoming persistent and chronic [9, 10]. Standardized instruments are vital for reliable patient assessment, enhancing clinical decisions and quality of care [13]. However, to date, there is no comprehensive questionnaire available in Dutch for early detection and evaluation of PFD within maternity care.

The German Pelvic Floor Questionnaire for Pregnant and Postpartum women (PFQ-PP), developed in 2017 by Metz et al. [14], provides a comprehensive assessment by addressing four domains of pelvic floor function: bladder function, bowel function, prolapse, and sexual health. Because it was developed specifically for pregnant and postpartum women, this questionnaire is an ideal foundation for cross-cultural validation in the Dutch context.

Therefore, the aim of this study was to translate, culturally adapt, and validate the German PFQ-PP for use in the Netherlands, resulting in the Dutch PFQ-PP.

## Materials and Methods

### Original German Pelvic Floor Questionnaire for Pregnant and Postpartum Women

The original Pelvic Floor Questionnaire (PFQ) was developed in Australia as an interviewer-administered pelvic floor questionnaire for research in community-dwelling women [15]. Later, it was adjusted and validated for use in clinical practice within a urogynecological population [16] and as an self-administered questionnaire [17]. In 2017, Metz et al. adjusted the German version of the PFQ into the German PFQ-PP, a self-administered pelvic floor questionnaire tailored specifically for pregnant and postpartum women [14]. The PFQ-PP covers four domains of pelvic floor function: bladder function (16 questions), bowel function (11 questions), prolapse (5 questions), and sexual health (9 questions). These 41 questions assess the presence, frequency, and severity of PFD symptoms, and their impact on quality of life (QoL). Additionally, Metz et al. included a newly developed domain for risk factors, and a postpartum module, which includes questions on the course of childbirth, emotional appraisal of the birth experience, and postpartum pain [14].

Most of the questions regarding the frequency, severity, and bothersomeness are assessed using a four-point Likert scale, except for questions regarding sexual activity, reasons for sexual abstinence, history of sexual trauma, sufficient lubrication, and type of dyspareunia on sexual intercourse, for which such scoring was found inappropriate. For these items, three-point Likert scales, yes/no, or categorical options were given. Each domain contains a response option “not applicable” for questions on bothersomeness. Domain scores are derived from the total of item scores within the domain, divided by the maximum score of the domain and multiplied by ten, resulting in a score between 0 and 10 per domain. The total score for pelvic floor function is the sum of these four domain scores (range 0–40), with a higher score indicating more/a higher degree of pelvic floor dysfunction. The maximum score for women who are not sexually active is 30. The highest scored response options are highlighted, to alert health care professionals to these concerns. The questionnaire is designed to assess symptoms associated with PFD used as an outcome; however, it is not intended to serve as a diagnostic tool. Therefore, it does not include a definitive cut-off score to indicate the presence of PFD. Instead, the scoring system is intended to monitor changes over time and evaluate the effectiveness of interventions.

### Type of Measurement Model

Measurement instruments for latent constructs are based on either reflective or formative models, distinguished by four criteria [18]: direction of causation, interchangeability of items, correlation between items, and the expectation of items to have the same antecedents and consequences. The studies on the development of the PFQ and PFQ-PP did not report on the type of measurement model (reflective or formative). The interpretation of the PFQ-PP as a formative model was based on the content of the items. Our author group determined that pelvic floor dysfunction and its subdomains are best measured using a formative model, and found the constructs measured within the PFQ-PP to be formative: direction of causality is from items to construct; items are not interchangeable; items do not necessarily correlate; and items do not necessarily have the same antecedents and consequences. In formative models, factor analysis and evaluation of its internal consistency are not of interest, as these analyses require reflective models [13]. Therefore, our study did not include these analyses in its quality assessment, as has been done in previous validations of the PFQ-PP [14, 19, 20].

### Study Design

This cross-sectional study involved the translation, cultural adaptation, and validation of the German PFQ-PP for use in the Netherlands. Permission to conduct the Dutch validation was obtained from the original authors. To ensure equivalence with the original German questionnaire, the translation and cultural adaptation process followed established guidelines for cross-cultural adaptation [21]. We used the COSMIN study design checklist [22] to design and conduct the psychometric evaluations.

#### Translation and Cultural Adaption

The questionnaire was independently forward-translated into Dutch by two translators: a native Dutch speaker and a native German speaker, both with excellent proficiency in the other language. Both translators were experienced midwives with professional knowledge of the concepts and topics covered in the questionnaire. Importantly, neither translator was familiar with the questionnaire prior to the translation process. Following the initial translations, a consensus meeting was held with the two translators and the research team. During this meeting, the two versions were synthesized into a single Dutch version by discussing and resolving discrepancies until consensus was reached. The wording was chosen to align with language level B1 of the Common European Framework of Reference [23], following the guidelines provided by Pharos, the Dutch Centre of Expertise on Health Disparities [24]. This synthesized version was then back-translated into German by a third translator, a native German speaker, with fluency in Dutch. The back-translator, a psychologist and researcher in midwifery science, possessed relevant subject knowledge but was not familiar with the questionnaire. The back-translated version was compared with the original questionnaire. No further adjustments were deemed necessary after the back-translation process.

#### Expert Review: Content Validity

An expert meeting of health care professionals was convened to review the latest version of the questionnaire, to assess its content validity, and to achieve consensus on maintaining semantic, linguistic, conceptual and cultural equivalence with the original German questionnaire. The expert committee included members with a diverse background: a professor of value-based maternity care, a professor of pelvis and pelvic-floor dysfunction, a urogynecologist, a researcher from the midwifery science department, and a medical student. In addition, an assistant professor from the department of medical decision making and two of the translators were present. A professor of general practice and a professor of urogynecology provided written feedback and suggestions prior to the meeting.

#### Pilot Test: Content Validity and Final Adaptation

The revised questionnaire underwent pre-testing through cognitive interviews. Participants were recruited via social media and the research department’s website, and received a financial compensation of 25 euros. Eligibility criteria were age 18 years or older, at least 28 weeks pregnant or between 6 weeks and 12 months postpartum, and fluent in Dutch. In total, 4 pregnant and 5 postpartum women were interviewed. During the interviews, content validity was assessed by exploring participants’ interpretation and understanding of each question-and-answer option. Participants were asked to explain the rationale behind their responses, and any signs of confusion or reluctance were explored by the interviewer through follow-up questions to provide clarification. The questionnaire was progressively refined based on the findings of the interviews. This process resulted in the final cross-culturally adapted Dutch version of the PFQ-PP.

#### Field Test: Participants and Data Collection

As there is no consensus on calculation of sample sizes in validation studies, we based our calculation on a practical guide to Measurement in Medicine [13], and comparable validation studies conducted in other languages [19, 20]. Based on this, the research team decided to recruit a minimum of 200 women, with at least 70 participants included in the re-test analysis. Inclusion criteria for women participating in the validation of the Dutch PFQ-PP were: age 18 years or older, pregnant with a gestational age of at least 28 weeks or between 6 weeks and 12 months postpartum, and sufficient proficiency in Dutch. Exclusion criteria included self-reported history of neurological disorders; malignancy; pelvic fractures; severe perianal skin disorders (e.g., lichen sclerosis); connective tissue disorders; pelvic surgery (except for surgical repair of childbirth injuries); or use of medications affecting bowel or bladder function.

Recruitment was conducted online via social media and the research department’s website from 20 to 29 December 2023. In addition to targeted advertising on social media, content creators on pregnancy and maternal health promoted the study on their social media profiles, and encouraged women to participate. The research department’s website provided detailed information about the study, eligibility criteria, contact details, and a portal for study enrolment. Upon enrolling, participants received a link to the digital questionnaire via email. The questionnaire was administered and coded using Castor EDC, where the study information was reiterated, and informed consent was obtained before starting the questionnaire. Inclusion and exclusion criteria were checked and socio-demographic data were collected. Women who did not meet the inclusion criteria or who met any exclusion criteria were unable to proceed to the questionnaire.

Participants were invited to complete the same questionnaire again after a 1-week interval to assess test–retest reliability and measurement error. A 1-week interval was chosen to minimize the influence of rapid physical changes occurring during pregnancy and the postpartum period, while also considering the risk of recall bias [25]. Intervals between 2 days and 2 weeks are generally considered appropriate for measuring test–retest reliability [26].

### Statistical Analyses

Only fully completed questionnaires were analyzed. Descriptive statistics were applied to describe the socio-demographic and obstetric characteristics of the participants, using numbers and percentages for nominal variables, and median and interquartile range (IQR) for continuous variables. These included age (years), educational level (low, intermediate, high), degree of urbanization (based on postal code: extremely urbanized, strongly urbanized, moderately urbanized, hardly urbanized, non-urbanized), ethnic background (Dutch, other), parity (number), obstetric history (ventouse, forceps, cesarean section, third- or fourth-degree perineal tear, weight of heaviest child at birth in grams), timing of completion of first questionnaire (gestational weeks or months since childbirth). Data were analyzed using SPSS version 28.0.1.1.

#### Test–Retest Reliability

Test–retest analysis was used to determine the extent to which the questionnaire produces consistent scores during repeated measurements, provided that the participants’ health remains stable. We calculated the intraclass correlation coefficient (ICC) for absolute agreement (i.e., ICC 2.1), and its corresponding 95% confidence interval (CI) [27]. The questionnaire was considered reliable if the obtained ICC values exceeded 0.70 [13].

#### Measurement Error of a Single Score

The measurement error of an instrument refers to the precision of the score, and is expressed by the standard error of measurement (SEM). The SEM can be used to provide a range around the observed score in which the theoretical “true” score lies. We calculated the SEM based on the variance estimates, using the following formula: SEM_agreement_ = √(σ^2^_m_ + σ^2^_residual_), where σ^2^_m_ is the variance due to systematic differences between moments of measurement, and σ^2^_residual_ for the residual variance [13].

#### Measurement Error of a Change Score

The measurement error of a change score refers to the uncertainty in the difference between two measurements (i.e., a change score) that is not due to actual changes in the underlying construct being measured, but instead arises from inaccuracies in the measurement process itself. The smallest detectable change (SDC) provides a value for the minimum change that must be observed in order to be confident (with at least 95%) that the observed change is real and not, potentially, a product of measurement error in the instrument. The SDC was calculated using the following formula: SDC = 1.96 * √(2) * SEM_agreement_ [13].

#### Known Group Validity

To establish construct validity, known group validity was evaluated by assessing the questionnaire’s ability to distinguish between women with and without pelvic floor symptoms. We hypothesized that women who suffered from pelvic floor symptoms in a specific domain would have at least 1.0-point higher domain scores than women who did not suffer from, or had no symptoms in, that domain. This is the minimal important difference (MID) that was described in the original German PFQ-PP [14]. “Suffering” was defined by the final question in each domain, which asked: “How much do your bladder/bowel/prolapse/sexual symptoms bother you?” Response options were: “not applicable—I have no symptoms,” “not at all,” “a little,” “quite a lot,” and “very much.” Participants responding with “a little,” “quite a lot,” or “very much” were classified as “suffering.”

## Results

The final version of the Dutch PFQ-PP can be found in Appendix A.

### Translation and Cultural Adaptation

During the process of translation and cultural adaption all semantic, linguistic, conceptual, and/or cultural discrepancies were discussed and resolved by consensus, resulting in slight modifications to the questionnaire, as explained below.

#### Overall Questionnaire

In order to achieve consistency in format and text across the questionnaire, answer options were re-arranged into a logical sequence. Some changes were made regarding the two final questions of each domain targeting QoL and bothersomeness. First, the questions were made consistent throughout the entire questionnaire and slightly rephrased to make them clearly refer to all preceding questions of the domain concerned. Second, as the direct Dutch translation of the German word “*Symptome*” (English: “symptoms”) resulted in “*symptomen*,” a word considered as medical jargon and rarely used by patients in the Netherlands, the expert committee decided to use the word “*klachten*” (English: “complaints”) instead.

During the pilot testing, women were unsure about what time period their answers should be based on. As a result, the time period they used as reference when completing the questionnaire varied markedly between the different questions and the women interviewed. An instruction was added, specifying a recall period of 4 weeks. This recall period was chosen based on a trade-off between reducing potential recall errors with a shorter recall period, and increasing included information with a longer recall period [28]. This time span also accounts for the rapid physical changes that occur during pregnancy and postpartum, as well as the variety of domains and symptoms covered in the questionnaire.

#### Bladder Function Domain

Two adjustments were made within the bladder function domain based on the pilot testing. Participants perceived the wording used for the different symptoms within question 7, regarding urinary stream, as similar and therefore confusing. The translation was adjusted and symptoms were described in more detail. Furthermore, a more concrete description of residual urine in question 9 was preferred, and the question was adapted accordingly.

#### Bowel Function Domain

Question 6 of the bowel domain was aligned to question 4 of the bladder function domain, as both questions addressed urge incontinence. The words “*plotseling*” (English: “sudden”) and “*meteen naar het toilet haasten*” (English: “rushing to the toilet”) were added, making the question more straightforward. For question 7 of the bowel function domain, the description of traces of fecal soiling was replaced by a Dutch word encompassing the same concept: “*remsporen*” (English: “skid marks”).

#### Prolapse Domain

The first question in the prolapse domain was reworded based on the formulation deemed most commonly used by health care professionals and patients in the Netherlands to describe the sensation and concept of prolapse, as determined by the expert committee [5].

#### Sexual Health Domain

The answer option “partner has problems/is impotent” for the second question of the sexual health domain was changed to “cause lies with partner” (Dutch: “*oorzaak bij partner*”) to make the question inclusive and applicable to all types of sexual orientation and behavior.

An explanatory note was added to the question “Are you sexually active?” to emphasize that “sexual activity” refers to the diverse array of activities of a sexual nature and not just sexual intercourse. Likewise, “*vrijen*” (German: “*des Verkehrs*”) was defined as “entering of something or someone into the vagina.” Taking into account women’s interpretation of a “neutral feeling” during intercourse, the response option “feel a lot” in question 2 was revised. In order to ensure sufficient contrast between all answer options, while aligning the response with a score of zero—indicating a neutral response with no increased risk of pelvic floor dysfunction—the option was changed to “feel enough.” Pilot testing confirmed that the revised option was clear and distinguishable to participants.

#### Postpartum Module

Participants in the pilot test were unsure whether “*bevallingen*” (English: “births”) included cesarean sections. The definition of “births” in the context of the PFQ-PP was therefore added, stating “all births at a gestational age of at least 16 weeks, including cesarean sections.”

The clarification “third- or fourth-degree perineal tear” was replaced by “*totaalruptuur*,” a commonly used Dutch term referring to both third- and fourth-degree perineal tears. The answer option “don’t know” was added for this question in case women were unsure whether this complication had occurred during (one of) their births. For question 6 of the postpartum module, regarding perineal pain, the answer option “yes” was divided into “yes—mild pain” and “yes—severe pain,” as it was assumed that the majority of women would experience some kind of perineal pain postpartum and that more information could be derived from the severity of pain, rather than just the presence of pain. For question 9 of the postpartum module, regarding feelings of anxiety during childbirth, an answer option “not applicable—had no anxiety” was added, because participants of the pilot test who had not experienced any anxiety during childbirth had difficulty answering this question.

### Study Population

**Fig. 1:**
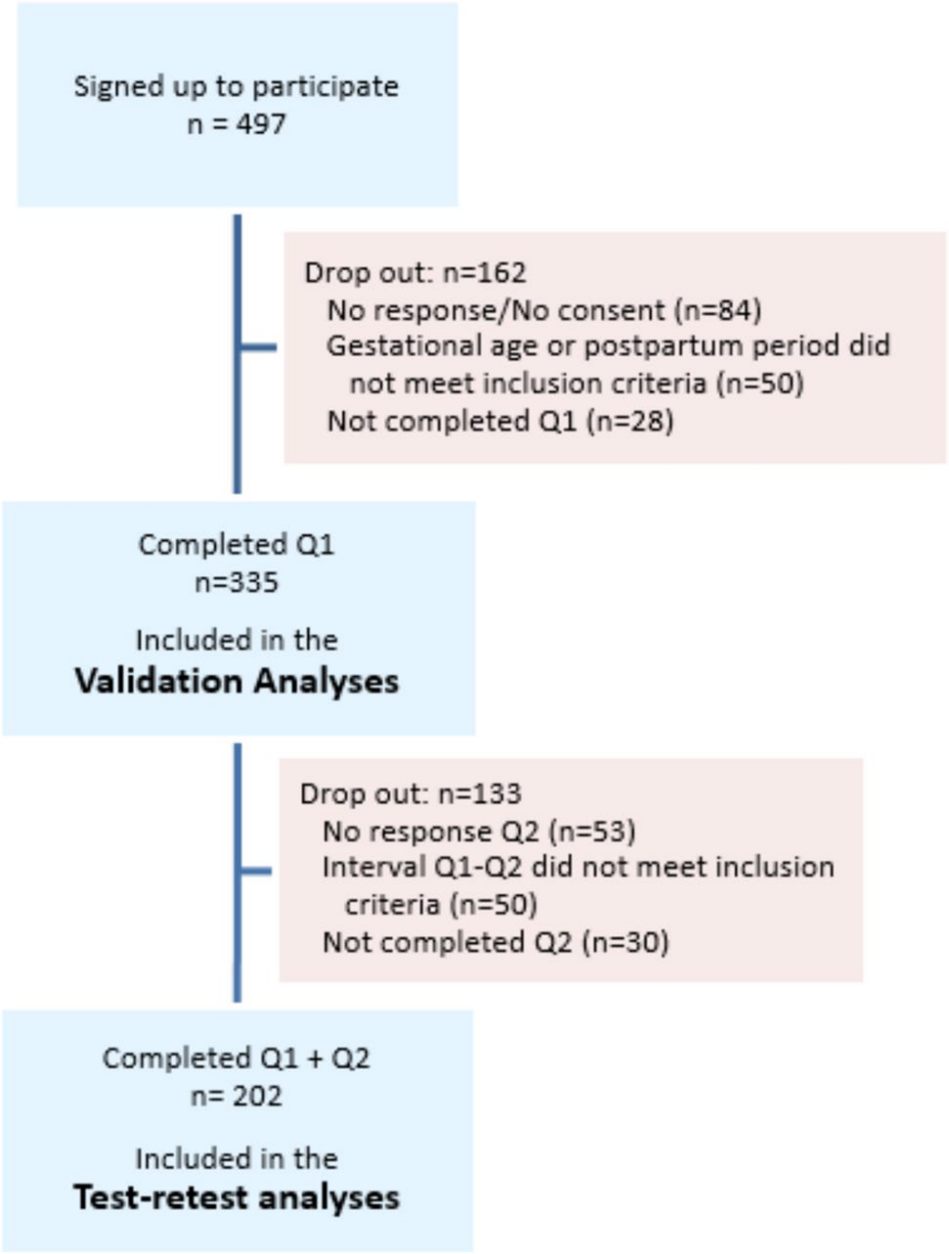
Flowchart of the study participants

A total of 497 women signed up to take part in the study, 84 of whom did not open the link to the questionnaire within the e-mail, or did not give full consent. No data are available on the characteristics of the women who did not open the questionnaire. Fifty women did not meet the inclusion criteria based on their pregnancy or postpartum duration. Another 28 women did not complete the first questionnaire: 17 women stopped during the questions on socio-demographic characteristics (prior to the start of the PFQ-PP), 4 women stopped after the bladder function domain, 1 stopped after the prolapse domain, 5 stopped after the sexual health domain, and 1 woman did not complete the postpartum module. In total, 335 participants were included in the validity and reliability analyses, including 87 women who were more than 28 weeks pregnant and 248 women who were between 6 weeks and 12 months postpartum. Of these women, 252 completed the second questionnaire. Among them, 47 participants completed the two questionnaires outside the designated 7- to 14-day interval and 3 participants gave birth between the first and second questionnaires, resulting in 202 participants who were included in the test–retest reliability analyses. The participant flow is shown in Fig. 1. The characteristics of the test–retest groups were comparable and are presented in Table 1.

**Table 1:** Participant characteristics in the validation and test–retest groups

Demographics	Validation sample, *N* = 335 (%)	Test–retest sample, *N* = 202 (%)
Age, years		
Median (IQR)	31 (29–34)	31 (29–34)
Educational level		
Low	2 (0.6)	0 (0)
Intermediate	73 (21.8)	45 (22.3)
High	260 (77.6)	157 (77.7)
Degree of urbanization	*n *= 333	*n *= 201
Extremely urbanized	47 (14.1)	31 (15.4)
Strongly urbanized	84 (25.2)	51 (25.4)
Moderately urbanized	54 (16.2)	35 (17.4)
Hardly urbanized	78 (23.4)	40 (19.9)
Non-urbanized	70 (21)	44 (21.9)
Ethnicity		
Dutch	330 (98.5)	200 (99.0)
Other	5 (1.5)	2 (1.0)
Parity		
0	35 (10.4)	22 (10.9)
1	152 (45.4)	93 (46.0)
2	120 (35.8)	70 (34.7)
3	22 (6.6)	13 (6.4)
4 or more	6 (1.8)	4 (2.0)
Obstetric history	*n*=300	*n*=180
Ventouse (1 or more)	45 (15)	28 (15.6)
Forceps (1 or more)	4 (1.3)	3 (1.7)
Cesarean section (one or more)	53 (17.7)	31 (17.2)
Third- or fourth-degree perineal tear (one or more)	20 (6.7)	12 (6.7)
Weight of heaviest child at birth, g		
Mean (SD)	3647 (487)	3642 (504)
< 2500 g	3 (1.0)	2 (1.1)
2500–3999 g	230 (76.7)	138 (76.7)
≥ 4000 g	67 (22.3)	40 (22.2)
Pregnant group	*n* = 87	*n* = 57
Gestational week at completion of first questionnaire		
28–33	42 (48.3)	27 (47.4)
34–39	41 (47.1)	30 (52.6)
40–41	4 (4.6)	0 (0)
Postpartum group	*n *= 249	*n *= 146
Months postpartum at completion of first questionnaire		
0–2	63 (25.3)	36 (24.7)
3–5	73 (29.3)	41 (28.1)
6–8	63 (25.3)	38 (26.0)
9–12	50 (20.1)	31 (21.2)

*IQR* interquartile range, *SD* standard deviation

### Psychometric Properties

#### Test–Retest Reliability

Intraclass correlation coefficients ranged from 0.82 to 0.92 for all domains and the total score, indicating good reliability (Table 2).

**Table 2:** Reliability, variance estimates, and measurement error of the Dutch Pelvic Floor Questionnaire for Pregnant and Postpartum women

Domain	ICC (95% CI)	σ^2^_p_	σ^2^_m_	σ^2^_residual_	SEM	SDC
Bladder	0.90 (0.86–0.93)	1.281	0.010	0.131	0.38	1.05
Bowel	0.82 (0.77–0.87)	1.127	0.009	0.232	0.49	1.36
Prolapse	0.90 (0.88–0.93)	3.339	0.000	0.356	0.60	1.80
Sexual	0.89 (0.86–0.92)	2.531	0.000	0.302	0.55	1.52
Total score	0.92 (0.89–0.94)	14.259	0.075	1.141	1.10	3.05

*ICC* intraclass correlation coefficient, σ^2^_p_ variance due to systematic differences between participants, σ^2^_m_ variance due to systematic differences between occasion, σ^2^_residual_ residual variance (i.e., random error variance), *SEM* standard error of measurement, *SDC* smallest detectable change

#### Standard Error of Measurement

The SEM ranged from 0.38 to 0.60 for the four domain scores (all ranging from 0 to 10). The SEM was 1.10 for the total score (range 0–40; Table 2).

#### Smallest Detectable Change

The SDC was 1.05 for the bladder function domain, 1.36 for the bowel function domain, 1.80 for the prolapse domain, and 1.52 for the sexual health domain. The SDC for the total score was 3.05 (Table 2).

#### Known Group Validity

The median domain scores of women who were suffering from their pelvic floor symptoms compared with those who did not were at least one point higher (MID) in all four domains (Table 3).

**Table 3:** Known group validity: domain scores for women with and without subjective suffering

Domain	Suffering	*n*	Median score (IQR)
Bladder	No	185	0.8 (0.4–1.5)
	Yes	150	2.5 (1.9–3.3)
Bowel	No	186	1.3 (0.6–1.6)
	Yes	149	2.6 (2.3–3.5)
Prolapse	No	257	0 (0–0)
	Yes	78	3.3 (2.0–6.0)
Sexual	No	173	0.4 (0–0.8)
	Yes	162	2.9 (2.1–4.2)

*IQR* interquartile range

## Discussion

The translated and cross-culturally adapted Dutch PFQ-PP showed good content validity and reliability. Psychometric properties were in accordance with the COSMIN criteria for good measurement properties [29] and similar to previous validation studies of the PFQ-PP [14, 19, 20]. This questionnaire can be used to assess the impact of symptoms and interventions on disease-specific quality of life during a particularly significant phase of life.

The Dutch PFQ-PP offers maternity care providers a validated instrument to objectify pelvic floor-related symptoms during pregnancy and postpartum. Standardized instruments in health care are essential to ensure valid, reliable, and responsive assessment of a patient’s health, which not only improves clinical decision making but also contributes to the overall quality of maternity care [13]. Pipitone and DeLancey emphasized the importance of addressing pelvic floor dysfunction during prenatal care, rather than waiting until women experience problems after childbirth [30]. Women often feel inadequately prepared for the physical changes that occur during the postpartum period and are hesitant to discuss pelvic floor issues owing to the stigma surrounding them [11, 12]. They would like care providers to actively inquire about PFDs [31]. Incorporating the PFQ-PP as a routine part of pre- and postnatal care in the Netherlands will potentially help to address this need. The PFQ-PP enables care providers to proactively assess and address pelvic floor issues, facilitating early detection and intervention, and ultimately improving maternal health outcomes by reducing the long-term impact of PFD. In addition to that, the instrument is highly valuable as it allows for monitoring of changes throughout the peripartum period and evaluation of the effectiveness of interventions. The self-administered nature of the PFQ-PP allows women to complete the questionnaire at their convenience. Responses indicating a high risk for pelvic floor issues are flagged with highlighted boxes, ensuring that health care providers are promptly alerted. This design makes the instrument user-friendly and efficient for both patients and health care professionals.

Acknowledging the PFQ as a formative model raises questions on the validity of domain scores and total score. We cannot assume that all items within a domain are equally important in contributing to a woman’s experience of impairment. For instance, women with daily accidental bowel leakage may experience far more inconvenience than women who need to strain daily to have a bowel movement, with an equal item score. Furthermore, it can be debated whether pelvic floor dysfunction is really a sum of the symptoms within each domain. Not every domain necessarily contributes equally to the subjective experience of pelvic floor dysfunction. Symptoms within one specific domain (for example, sexual health) may be far more acceptable to women than symptoms within another domain (for example, bowel function). We advise health care providers to focus on the individual item scores when using the PFQ-PP as a screening instrument. Total and domain scores may be of interest when used to assess progress, and should be interpreted with caution. Further research is needed.

Reporting the SEM and the SDC was considered important for gaining insight into their reliability and error around the observed domain or total score. For example, the SEM for the bladder function domain (with a score range of 0–10) was found to be 0.38, which means in 95% of cases, a woman’s true score will be between the observed score plus and minus 0.74 (i.e., 1.96 * SEM). An SDC of 1.05 within the bladder function domain suggests that score differences smaller than 1.05 in this domain might be due to chance. These parameters are particularly useful for interpreting the scores in clinical practice. However, a patient-relevant minimal important change score has not yet been established for the PFQ-PP, underscoring the importance of addressing this in future research.

### Strengths and Limitations

Adjustments made after pilot testing improved the clarity of the questionnaire. However, these changes may have slightly reduced its comparability with the original German scale. Nonetheless, they were aimed at maximizing the content validity of the Dutch version. These adjustments could be incorporated into updates of the original German PFQ-PP and considered for new translations of the PFQ-PP. A recall period was added to the questionnaire, to emphasize that answers should be based on experiences in the past 4 weeks. Adding an adequate recall period to the questionnaire improved the accuracy of self-reported pelvic floor symptoms and thus the reliability and validity. Similar recall periods for self-reported questionnaires covering pelvic floor function have been reported [32, 33].

A further strength of our study is the relatively large sample size of 335 women, 202 of whom were included in the test–retest analysis. Furthermore, participants’ characteristics showed a wide distribution in terms of age, degree of urbanization, gestational weeks, and postpartum months. Unfortunately, educational level and ethnicity were quite homogenous within our study population, as the majority of participants were highly educated and of Dutch ethnicity. A possible contributing factor could have been our recruitment through social media. The content creators who collaborated with our study were predominantly white women, who created pregnancy and maternal health-related content. Although they collectively had over 140,000 followers, which expanded our reach considerably, the demographic diversity of these followers may have been limited. This may affect the generalizability of our findings to broader socio-demographic contexts, as comprehension and interpretation of the questionnaire may differ between ethnic groups and educational levels. In addition, self-selection bias may have reduced the representativeness of our study, as women who experienced pelvic floor symptoms may have been more likely to participate [34]. Consequently, symptomatic women were overrepresented in our study population. For example, in our study the prevalence of UI during the third trimester of pregnancy was 67%, compared with 47% in previous studies [7]. Furthermore, our study contained substantially more women reporting to suffer compared with the German study. Even though our study population may not be fully representative of the final target population, the large number of symptomatic women resulted in a wide range of reported answers, allowing a more precise validation and reliability assessment for this particular group.

The retest questionnaire was sent at a fixed 1-week interval, but, owing to technical limitations, women were not restricted to completing the questionnaire on the same day on which they received the email containing the link to the questionnaire. As a result, the interval between completion of the two questionnaires ranged from 0 to 21 days. To ensure a reliable test–retest interval, we only included women who filled out the questionnaire within the 7- to 14-day interval. However, the German study used a 1-day interval, potentially increasing the risk of participants recalling their responses from the initial questionnaire. The longer test–retest interval used in our study could have affected the test–retest reliability results.

Our study did not perform an analysis of responsiveness. Future research should include longitudinal follow-up of participants to evaluate the responsiveness of the questionnaire. In addition, criterion validity was not determined as no gold standard is available. Future studies should consider comparison with other validated questionnaires focusing on the separate domains, to determine convergent validity. Additionally, structural validity of the PFQ-PP could be determined in future research, for example, by examining the items as predictive variables. This may help to identify whether certain items carry more weight in representing the construct and should therefore contribute more significantly to the overall score.

## Conclusion

The German PFQ-PP has been successfully translated and culturally adapted for use in the Netherlands. The self-administered Dutch PFQ-PP is valid and reliable for assessing bladder function, bowel function, prolapse, and sexual health in pregnant and postpartum women. The questionnaire provides significant value in the proactive assessment of pelvic floor dysfunction, effectively addressing women’s care needs related to pelvic floor health during pregnancy and the postpartum period. The PFQ-PP is an efficient and user-friendly tool, making it well-suited to routine integration into pre- and postnatal care practices. Further research is needed to determine the responsiveness of the Dutch PFQ-PP.

## Supplementary Information

Below is the link to the electronic supplementary material.

Supplementary file1 (DOCX 65 KB)

## Data Availability

The coded data that support the findings of this study are available from the corresponding author upon request and may be shared for research purposes only.
