# Supplementary material for: Dutch Translation, Cultural Adaption, and Validation of the German Pelvic Floor Questionnaire for Pregnant and Postpartum Women
Source: Int Urogynecol J. 2025 Jun 3;36(11):2229–39. doi: 10.1007/s00192-025-06173-3 (PMC12681456; doi:10.1007/s00192-025-06173-3)
Supplement: Supplementary file 1 — Supplementary file1 (DOCX 65 KB) [file 192_2025_6173_MOESM1_ESM.docx]

**Appendix A**

**Bekkenbodem vragenlijst ‘PFQ-PP’**Tijdens zwangerschap en na de bevalling

Scorelijst

**Risicofactoren**

| Lengte  ��� cm | Gewicht ��� kg | Gewicht voor de zwangerschap ��� kg | BMI ��, � | | >25 |
| --- | --- | --- | --- | --- | --- |
| Leeftijd | | | �� Jaar | | >35 |
| Zijn er aan uw kant van de familie vrouwen die problemen hebben met het ophouden van urine of van ontlasting, of die last hebben van verzakkingen? | | | nee | weet ik niet | ja |
| Rookt u? | | | nee | ben gestopt | ja |
| Kunt u uw bekkenbodemspieren gericht aanspannen? | | | ja | weet ik niet | nee |

**Blaasfunctie**

| 1. Hoe vaak moet u overdag plassen? | elke 3 uur = 0 | elke 2 uur = 1 | | 1 keer per uur = 2 | | vaker = 3 | |
| --- | --- | --- | --- | --- | --- | --- | --- |
| 2. Hoe vaak wordt u ’s nachts wakker omdat u moet plassen? | 0-1 keer = 0 | 2 keer = 1 | | 3 keer = 2 | | vaker dan  3 keer = 3 | |
| 3. Verliest u urine tijdens het slapen? | nooit = 0 | soms – minder dan 1 keer per week = 1 | | vaak – 1 keer of vaker per week = 2 | | bijna altijd - dagelijks = 3 | |
| 4. Hoe vaak is de aandrang om te plassen zo sterk, dat u meteen naar het toilet moet haasten? | nooit = 0 | soms – minder dan 1 keer per week = 1 | | vaak – 1 keer of vaker per week = 2 | | bijna altijd - dagelijks = 3 | |
| 5. Verliest u bij plotseling sterke aandrang urine voordat u het toilet heeft bereikt? | nooit = 0 | soms – minder dan 1 keer per week = 1 | | vaak – 1 keer of vaker per week = 2 | | bijna altijd - dagelijks = 3 | |
| 6. Verliest u urine tijdens het hoesten, niezen, lachen, tillen of sporten? | nooit = 0 | soms – minder dan 1 keer per week = 1 | | vaak – 1 keer of vaker per week = 2 | | bijna altijd - dagelijks = 3 | |
| 7. Is uw urinestraal zwak, duurt het lang voordat de urinestraal op gang komt of voordat u bent uitgeplast? | nooit = 0 | soms – minder dan 1 keer per week = 1 | | vaak – 1 keer of vaker per week = 2 | | bijna altijd - dagelijks = 3 | |
| 8. Kunt u goed inschatten hoe vol uw blaas is? | ja – altijd = 0 | meestal = 1 | | soms = 2 | | nee – nooit = 3 | |
| 9. Heeft u het gevoel dat u niet helemaal kunt uitplassen? | nooit = 0 | soms – minder dan 1 keer per week = 1 | | vaak – 1 keer of vaker per week = 2 | | bijna altijd - dagelijks = 3 | |
| 10. Moet u druk zetten om te kunnen plassen? | nooit = 0 | soms – minder dan 1 keer per week = 1 | | vaak – 1 keer of vaker per week = 2 | | bijna altijd - dagelijks = 3 | |
| 11. Draagt u inlegkruisjes of verband voor urineverlies? | nooit = 0 | soms - uit voorzorg = 1 | | vaak – tijdens het sporten/bij verkoudheid = 2 | | bijna altijd - dagelijks = 3 | |
| 12. Drinkt u bewust minder om urineverlies te voorkomen? | nooit = 0 | soms – minder dan 1 keer per week = 1 | | vaak – 1 keer of vaker per week = 2 | | bijna altijd - dagelijks = 3 | |
| 13. Heeft u een brandend, trekkend of pijnlijk gevoel tijdens het plassen? | nooit = 0 | soms – minder dan 1 keer per week = 1 | | vaak – 1 keer of vaker per week = 2 | | bijna altijd - dagelijks = 3 | |
| 14. Hoe vaak heeft u een blaasontsteking? | zelden of nooit = 0 | 1-3 keer  per jaar = 1 | | 4-12 keer  per jaar = 2 | | 1 keer of vaker per maand = 3 | |
| 15. Belemmeren deze klachten uw dagelijkse leven? (bijvoorbeeld tijdens sport, werk, boodschappen doen, uitgaan) | niet van toepassing – heb geen klachten = 0 | helemaal niet = 0 | een beetje = 1 | | nogal = 2 | | heel erg = 3 |
| 16. Hoe vervelend vindt u uw blaasklachten? | niet van toepassing – heb geen klachten = 0 | helemaal niet = 0 | een beetje = 1 | | nogal = 2 | | heel erg = 3 |

**Darmfunctie**

| 1. Hoe vaak heeft u ontlasting? | meerdere keren per dag = 1 | dagelijks tot elke 3 dagen = 0 | | elke  4-7 dagen = 1 | | minder dan  1 keer per week = 2 | |
| --- | --- | --- | --- | --- | --- | --- | --- |
| 2. Wat is de gewoonlijke vastheid van uw ontlasting? | verschillend = 1 | heel hard = 1 | | zacht of gevormd = 0 | | dun/brijig = 2 | |
| 3. Perst u heel hard bij ontlasting? | nooit = 0 | soms – minder dan 1 keer per week = 1 | | vaak – 1 keer of vaker per week = 2 | | bijna altijd - dagelijks = 3 | |
| 4. Heeft u last van verstopping (obstipatie)? | nooit = 0 | soms – minder dan 1 keer per week = 1 | | vaak – 1 keer of vaker per week = 2 | | bijna altijd - dagelijks = 3 | |
| 5. Laat u windjes zonder dat u dat kunt tegenhouden? | nooit = 0 | soms – minder dan 1 keer per week = 1 | | vaak – 1 keer of vaker per week = 2 | | bijna altijd - dagelijks = 3 | |
| 6. Hoe vaak krijgt u plotseling zo’n sterke aandrang voor ontlasting, dat u meteen naar het toilet moet haasten? | nooit = 0 | soms – minder dan 1 keer per week = 1 | | vaak – 1 keer of vaker per week = 2 | | bijna altijd - dagelijks = 3 | |
| 7. Ziet u “remsporen” in uw onderbroek of verband? | nooit = 0 | soms – minder dan 1 keer per week = 1 | | vaak – 1 keer of vaker per week = 2 | | bijna altijd - dagelijks = 3 | |
| 8. Verliest u ongewild ontlasting? | nooit = 0 | soms – minder dan 1 keer per week = 1 | | vaak – 1 keer of vaker per week = 2 | | bijna altijd - dagelijks = 3 | |
| 9. Heeft u het gevoel dat u uw darmen niet helemaal kunt legen? | nooit = 0 | soms – minder dan 1 keer per week = 1 | | vaak – 1 keer of vaker per week = 2 | | bijna altijd - dagelijks = 3 | |
| 10. Belemmeren deze klachten uw dagelijkse leven? (bijv. dagplanning, sport, werk, boodschappen doen, uitgaan) | niet van toepassing –heb geen klachten = 0 | helemaal niet = 0 | een beetje = 1 | | nogal = 2 | | heel erg = 3 |
| 11. Hoe vervelend vindt u uw darmklachten? | niet van toepassing – heb geen klachten = 0 | helemaal niet = 0 | een beetje = 1 | | nogal = 2 | | heel erg = 3 |

**Verzakking**

| 1. Voelt of ziet u een bal in of uit uw vagina? | nooit = 0 | soms – minder dan 1 keer per week = 1 | | vaak – 1 keer of vaker per week = 2 | | meestal - dagelijks = 3 | |
| --- | --- | --- | --- | --- | --- | --- | --- |
| 2. Heeft u het gevoel dat uw vagina of baarmoeder is verzakt? | nooit = 0 | soms – minder dan 1 keer per week = 1 | | vaak – 1 keer of vaker per week = 2 | | meestal - dagelijks = 3 | |
| 3. Heeft u het gevoel dat uw vagina of baarmoeder tijdens het tillen, lopen of rennen zakt? | helemaal niet = 0 | een beetje = 1 | | nogal = 2 | | heel erg = 3 | |
| 4. Belemmeren deze klachten uw dagelijkse leven? (bijv. dagplanning, sport, werk, boodschappen doen, uitgaan) | niet van toepassing – heb geen klachten = 0 | helemaal niet = 0 | een beetje = 1 | | nogal = 2 | | heel erg = 3 |
| 5. Hoe vervelend vindt u uw verzakkingsklachten? | niet van toepassing – heb geen klachten = 0 | helemaal niet = 0 | een beetje = 1 | | nogal = 2 | | heel erg = 3 |

**Seksleven**

| Bent u seksueel actief?  (Denk hierbij aan alle vormen van seksuele activiteit: bijvoorbeeld strelen, voorspel, masturbatie en geslachtsgemeenschap). | helemaal niet | | zelden | | | | regelmatig | | |
| --- | --- | --- | --- | --- | --- | --- | --- | --- | --- |
| Wat is de reden dat u niet seksueel actief bent? | geen partner | oorzaak ligt bij partner | | | voel geen opwinding  of lust | | | seks vind ik onaangenaam omdat ………. | |
| Heeft u een nare seksuele ervaring meegemaakt, waar u nu nog veel last van heeft? | nee | | | | ja | | | | |
| Bij de volgende vragen wordt met ‘vrijen’ bedoeld: het binnengaan van iets of iemand in de vagina. | | | | | | | | | |
| 1. Wordt uw vagina voldoende vochtig tijdens het vrijen? | ja = 0 | | | | nee = 1 | | | | |
| 2. Hoe is het gevoel in uw vagina tijdens het vrijen? | ik voel voldoende = 0 | ik voel weinig = 1 | | | ik voel niets = 2 | | | ik voel pijn = 3 | |
| 3. Denkt u dat uw vagina te slap of te wijd is? | nee - nooit = 0 | soms = 1 | | | vaak = 2 | | | altijd = 3 | |
| 4. Denkt u dat uw vagina te strak of te nauw is? | nee - nooit = 0 | soms = 1 | | | vaak = 2 | | | altijd = 3 | |
| 5. Heeft u pijn tijdens het vrijen? | nee - nooit = 0 | soms = 1 | | | vaak = 2 | | | altijd = 3 | |
| 6. Indien u pijn heeft tijdens het vrijen, waar zit de pijn? | bij de vagina ingang = 1 | | diep van binnen/  in het bekken = 1 | | | | beide = 2 | | |
| 7. Verliest u ongewild urine of ontlasting tijdens seksuele activiteiten? | nee - nooit = 0 | soms = 1 | | | vaak = 2 | | | altijd = 3 | |
| 8. Belemmeren deze klachten uw seksleven? | niet van toepassing - heb geen klachten = 0 | helemaal niet = 0 | | een beetje = 1 | | nogal = 2 | | | heel erg = 3 |
| 9. Hoeveel last heeft u van uw seksuele klachten? | niet van toepassing - heb geen klachten = 0 | helemaal niet = 0 | | een beetje = 1 | | nogal = 2 | | | heel erg = 3 |

**Na de bevalling**

| Hoe vaak bent u bevallen? (hieronder vallen alle bevallingen na een zwangerschapsduur van minimaal 16 weken, inclusief keizersnedes). | �� | Bij hoeveel bevallingen werd een zuignap/vacuümpomp gebruikt? | | | | | | �� |
| --- | --- | --- | --- | --- | --- | --- | --- | --- |
| Bij hoeveel bevallingen is er een keizersnede gedaan? | �� | Bij hoeveel bevallingen werd een verlostang gebruikt? | | | | | | �� |
| Hoeveel woog uw zwaarste kind bij de geboorte? | | ���� g | | | | | | >4000 g |
| Heeft u tijdens een van uw bevallingen een beschadiging aan uw kringspier/anus of darmen (totaalruptuur) gehad? | | nee | | ja | | | weet ik niet | |
| Had u na de bevalling pijn in het gebied van de vagina, het perineum of de kringspier/anus? | | nee | | ja – lichte pijn | | | ja – erge pijn | |
| Heeft u het gevoel dat u de bevallingspijn en de pijn na de bevalling heeft verwerkt? | | ja | grotendeels | | een   beetje | | | nee |
| Heeft u het gevoel dat u de angsten, die u tijdens de bevalling had, heeft verwerkt? | | ja | grotendeels | | een   beetje | nee | | niet van   toepassing -  geen angsten   gehad |

**Score**

| **Blaasfunctie** | Vraag 1-16 | Score / 48 | = . | x 10 = |  |
| --- | --- | --- | --- | --- | --- |
| **Darmfunctie** | Vraag 1-11 | Score / 31 | = . | x 10 = |  |
| **Verzakking** | Vraag 1-5 | Score / 15 | = . | x 10 = |  |
| **Seksleven** | Vraag 1-9 | Score / 24 | = . | x 10 = |  |
| **Blaas score + Darm score + Verzakking score + Seksleven score =** | | | |  |  |
